# Intranasal application of polyethyleneimine suppresses influenza virus infection in mice

**DOI:** 10.1038/emi.2016.64

**Published:** 2016-04-27

**Authors:** Biao He, Yuhong Fu, Shuai Xia, Fei Yu, Qian Wang, Lu Lu, Shibo Jiang

**Affiliations:** 1 Key Lab of Medical Molecular Virology of MOE/MOH, School of Basic Medical Sciences and Shanghai Public Health Clinical Center, Fudan University, Shanghai 200032, China; 2 New York Blood Center, Lindsley F. Kimball Research Institute, New York, NY 10065, USA

**Dear Editor**,

Emerging and reemerging viruses that cause respiratory infectious diseases, such as severe acute respiratory syndromes coronavirus (SARS-CoV), Middle East respiratory syndrome coronavirus (MERS-CoV), and influenza viruses, are a significant threat to public health worldwide. Although vaccines are the most effective strategy to prevent viral infection, vaccine development is a long process and may be effective only against the corresponding virus. Therefore, it is essential to develop antiviral agents for intranasal administration as nonspecific prophylaxis against infection by emerging or reemerging respiratory viruses with epidemic or pandemic potential.

Cholera toxin (CT), which acts as a mucosal adjuvant to stimulate mucosal and systemic immune responses, has the potential for intranasal application as an immunotherapeutic against infection by respiratory viruses.^1^ However, CT can exacerbate lung pathology after influenza virus infection through the induction of IL-17, a potent proinflammatory cytokine, because co-administration of an IL-17RA neutralizing antibody with CT attenuates lung pathology and increases protection against influenza virus infection.^2^ Therefore, we hypothesized that intranasal application of a mucosal stimulant that induces antiviral cytokines, except IL-17, may be effective against influenza virus infection.

In this regard, polyethyleneimine (PEI), a mucosal adjuvant, exhibits stronger mucosal adjuvanticity but induces much lower IL-17 expression than cholera toxin subunit B (CTB).^3^ Therefore, for the first time, we tested PEI for its potential protective effects against influenza virus infection. Ten-week-old specific-pathogen-free (SPF) female Balb/c mice were anesthetized with pelltobarbitalum natricum. Then, 50 μL of PBS containing 20 μg of 25 kD linear PEI (Polysciences, Warrington, PA) or 2.5 μg of CTB (Sigma-Aldrich, St Louis, MO, USA) or PBS alone as a control was intranasally administered twice at 24 and 48 h before challenge with 5 LD_50_ influenza virus H1N1 (A/PR/8/34). Mouse body weights were monitored every day, and those with greater than 25% loss of their initial body weight were euthanized as described.^4^

As shown in Figure 1A, the body weight of the mice in the PEI-pretreated group remained stable from day 1 to day 5 after H1N1 challenge and then gradually decreased until day 11, for a total loss of 18%, before recovering. By contrast, the body weights of the mice in the CTB- and PBS-pretreated groups decreased significantly beginning on day 2 and reached losses of more than 25% by day 8 and 10, respectively, after H1N1 challenge. The final survival rate of the mice in the PEI-pretreated group was 60%, whereas that of the mice in the CTB- and PBS-pretreated groups was 0% (Figure 1B). We then examined the viral titers in mouse lungs as previously described^5^ and found that PEI significantly reduced lung viral titers on day 2 after H1N1 challenge, whereas the viral titers in the lungs of mice in the CTB- and PBS-pretreated groups showed no significant differences (Figure 1C). Subsequently, we examined lung sections stained with hematoxylin and eosin as previously described.^2^ On day 2 after H1N1 infection, the pulmonary alveoli were relatively intact, and only a few inflammatory cells were observed in the lung tissues of mice in the PEI-pretreated group. However, the lungs of mice in the CTB- and PBS-pretreated groups were filled with abundant inflammatory cells (Figure 1D). These results suggest that intranasal application of PEI has efficacy in protecting mice from challenge by influenza virus H1N1.

To determine the efficacy of PEI against another subtype of influenza virus, we pretreated mice with PBS containing PEI or PBS alone and challenged them with 5 LD_50_ influenza virus H3N2 (A/Guizhou/54/89). Mice in the PEI-pretreated group were fully protected against H3N2 challenge, showing no significant body weight loss (Figure 1E) and a 100% survival rate, whereas those in the PBS group lost more than 25% of their body weight and showed a 0% survival rate on day 6 post-challenge (Figure 1F). Therefore, PEI-mediated protection against influenza virus infection is not subtype-specific.

To elucidate the mechanism of action of PEI, we examined the RNA levels of IFN-α4, IFN-β, IFN-γ, GM-CSF, IFITM3 and IL-17 in the lungs of mice pretreated with PEI, CTB, and PBS, respectively, before viral challenge using quantitative reverse transcription-PCR (qRT-PCR). As previously reported, some of these cytokines, such as interferon, GM-CSF and IFITM3, were effective in protecting against influenza infection.^6, 7, 8^ As shown in Supplementary Figure S1, PEI induced a significantly higher RNA level of IFN-α4 than CTB or PBS. Furthermore, the RNA levels of GM-CSF and IFITM3 elicited by PEI were similar to those induced by CTB but much higher than those induced by PBS. The RNA level of IL-17 in mice pretreated with CTB, an IL-17-inducing adjuvant, was approximately 7- and 42-fold higher than that in mice pretreated with PEI and PBS, respectively. These results suggest that IFN-α4, GM-CSF, and IFITM3 are ‘good cytokines' because they act as protective mediators against influenza virus infection, whereas IL-17 is a ‘bad cytokine' that exacerbates pathology, primarily in the lung, after influenza infection.

In summary, we demonstrated that PEI, a mucosal stimulant for topical intranasal administration, is highly effective in preventing influenza virus infection. Compared to the bacteria-produced toxin CTB, the chemically synthesized polymer PEI is safer for mucosal application in humans. PEI has been tested in several clinical trials for gene delivery *in vivo*, demonstrating a good safety profile.^9^ Moreover, its low cost of production and abundance makes PEI more suitable for urgent and widespread use during a time of influenza epidemic or pandemic.

## Figures and Tables

**Figure 1:**
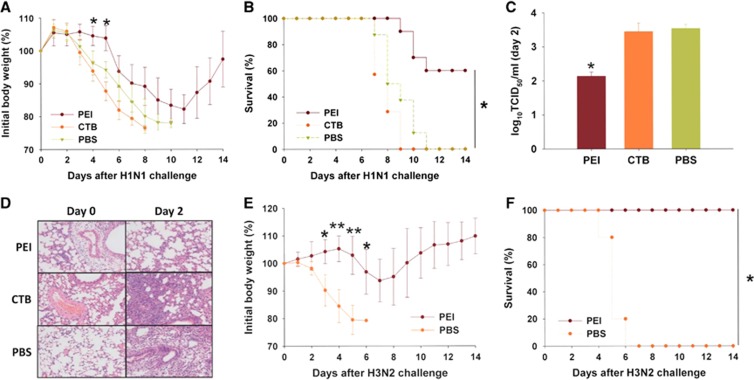
Protective effects of PEI against H1N1 and H3N2 challenge. (**A**) Body weight loss after 5LD_50_ H1N1 challenge. (**B**) Survival rate of mice in the PEI-pretreated group (*n*=10), CTB-pretreated group (*n*=7), and PBS-pretreated group (*n*=8) after 5 LD_50_ H1N1 challenge. (**C**) Lung virus titer on day 2 after 5 LD_50_ H1N1 challenge. For panels A to C, *a significant difference (*P<0.05) was observed between the PEI-pretreated group and the CTB- as well as PBS-pretreated groups. (**D**) Examination of lung pathology in lung tissue sections stained with hematoxylin and eosin. Original magnification: × 200. (**E**) Body weight loss of mice after 5 LD_50_ H3N2 challenge. (**F**) Survival rate of mice after 5 LD_50_ H3N2 challenge. For panels E and F, *a significant difference (*P<0.05) and **very significant difference (**P<0.01) were observed between the PEI- and PBS-pretreated groups. Data are expressed as means±SD. All results were repeated and verified at least twice.
