# Supplementary material for: Intranasal application of polyethyleneimine suppresses influenza virus infection in mice
Source: Emerg Microbes Infect. 2016 Apr 27;5(4):e41–. doi: 10.1038/emi.2016.64 (PMC4855075; doi:10.1038/emi.2016.64)
Supplement: Supplementary Figure S1 [file emi201664x1.pdf]

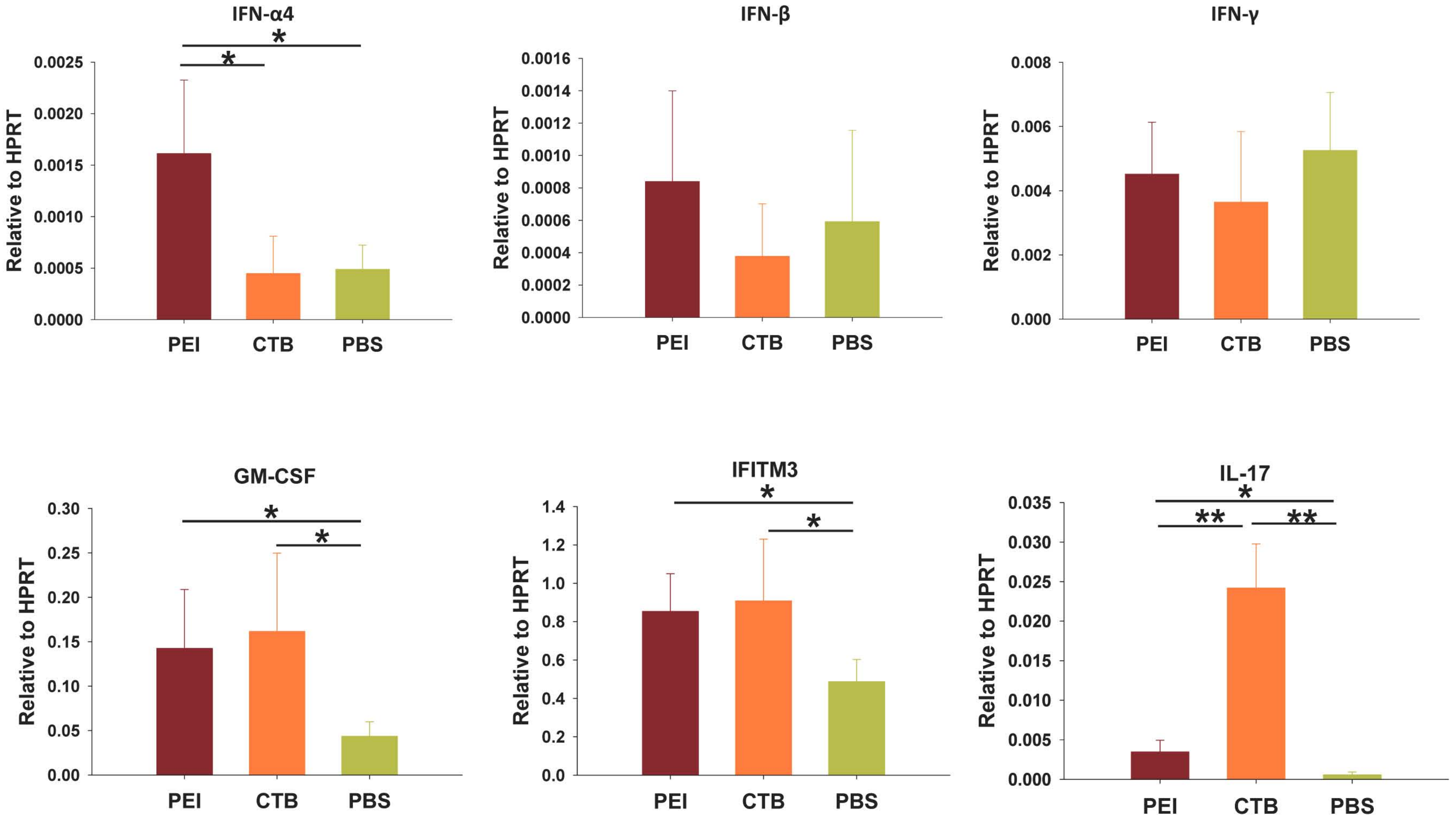

Supplementary Figure S1 RNA levels of cytokines in mice intranasally pretreated with PEI, CTB and PBS, respectively. Lungs were retrieved after pretreatment and before influenza virus challenge for RNA quantification using qRT-PCR. Data are representative of 2 independent experiments with four mice in each group. HPRT is hypoxanthine phosphoribosyltransferase. *Significant difference (*P<0.05) and **very significant difference (**P<0.01).
